# Treating deep venous insufficiency with a novel implantable device

**DOI:** 10.1016/j.jvscit.2024.101554

**Published:** 2024-06-21

**Authors:** Mark Awad, Nicholas Schaper, Saideep Bose, Matthew R. Smeds

**Affiliations:** a Saint Louis University Division of Vascular and Endovascular Surgery, St. Louis, MO; b Saint Louis University School of Medicine, St. Louis, MO

**Keywords:** Venous Insufficiency, Implantable device, Vein, Valve, Treatment

## Abstract

Chronic venous insufficiency (CVI) is increasing in prevalence on a global scale. Current treatment options are limited to improving venous return, ablation of refluxing veins, and reducing outflow obstruction. A new bioprosthetic device, the VenoValve, may bridge the gap of treatment for patients with chronic venous insufficiency who have failed prior treatment. We demonstrate the treatment of a 72-year-old man with bilateral venous insufficiency and leg wounds using this device in his left femoral vein via an open anterior surgical approach. The patient had no postoperative complications, and a patent valve at 6 months. The VenoValve may be a viable option for patients with advanced chronic venous insufficiency.

Chronic venous insufficiency (CVI), a subset of chronic venous disease, is a pathology increasing in prevalence with rates reported as high as 40% in women and 17% in men on a global scale.^1^ CVI develops as a result of the failure of the bicuspid valves located within the peripheral venous system that aid in the return of blood to the heart; this condition affects the lower extremities primarily, where gravity has its greatest effect in bipedal organisms.^1^ CVI is classified according to the Clinical-Etiological-Anatomical-Pathophysiological scale, which includes clinical evidence of severe deep venous reflux ranging from telangiectasia to venous leg ulcers (Table).^2^ Complications of CVI include deep venous thrombosis, increased risk of infection, pain, and leg ulceration.^3^ This disease can greatly impact a patient's quality of life, cause financial stress, and places a burden on healthcare resources.^3^^,^^4^ More than two million patients per year suffer from venous ulceration with approximately 150,000 new patients diagnosed with chronic venous disease in the United States each year, yet available treatment options for this population have historically been limited.^3^^,^^5^ Currently, treatment focuses on improving venous return, treating superficial reflux, and decreasing outflow obstruction. This goal is typically accomplished with medical management including compression therapy, leg elevation, and exercise programs for conservative management, and procedures such as superficial endovenous ablation, saphenous vein ligation and stripping, or venoplasty and stenting of outflow iliac lesions.^1^

**Table:** Clinical-Etiological-Anatomical-Pathophysiological clinical (c) classifications

C Class	Description
C0	No visible or palpable signs of venous disease
C1	Telangiectasias or reticular veins
C2	Varicose veins
C2r	Recurrent varicose veins
C3	Edema
C4	Changes in skin and subcutaneous tissue secondary to CVD
C4a	Pigmentation or eczema
C4b	Lipodermatosclerosis or atrophie blanche
C4c	Corona phlebectatica
C5	Healed venous ulcer
C6	Active venous ulcer
C6r	Recurrent active venous ulcer

*CVD*, Chronic venous disease.

Each clinical class subcharacterized by a subscript indicating the presence (symptomatic, s) or absence (asymptomatic, a) of symptoms attributable to venous disease.

Adapted from Lurie et al.

However effective, these treatments predominantly improve superficial venous flow without significant deep venous impact, leaving few options for patients with advanced stage disease.^3^ Several surgical options for correction of CVI have been described as early as 1960, with none showing effective treatment in long-term follow-up, leaving no accepted standard surgical options for these patients.^3^ Previous approaches have involved direct surgical revision of incompetent valves with valvuloplasty, venous banding, or valve transplantation.^6^ A new device, the VenoValve (enVVeno Medical Corporation, Irvine, CA), has undergone first-in-man studies and recently completed enrollment in the U.S. pivotal trial (Surgical Anti-reflux Venous Valve Endoprosthesis [SAVVE] Study) and may bridge the gap of treatment for patients with CVI who have failed prior treatment.^4^^,^^7^ The VenoValve is a bioprosthetic monocuspid valve composed of a porcine aortic leaflet (nonantigenic, antithrombogenic) sewn into a stainless-steel frame (Fig).^3^ This valve is meant to be implanted surgically into the deep venous system at the femoral vein in the mid-thigh position. The device has been shown to significantly improve reflux time in the popliteal vein, disease severity (Revised Venous Clinical Severity Score), and visual analogue scale pain scores at the 2-year follow-up.^3^ We present a video of a gentleman with C6 venous insufficiency who received implantation of the VenoValve device in his left femoral vein via an open anterior approach. The patient provided written informed consent for the report of his case details and imaging studies.

**Fig:**
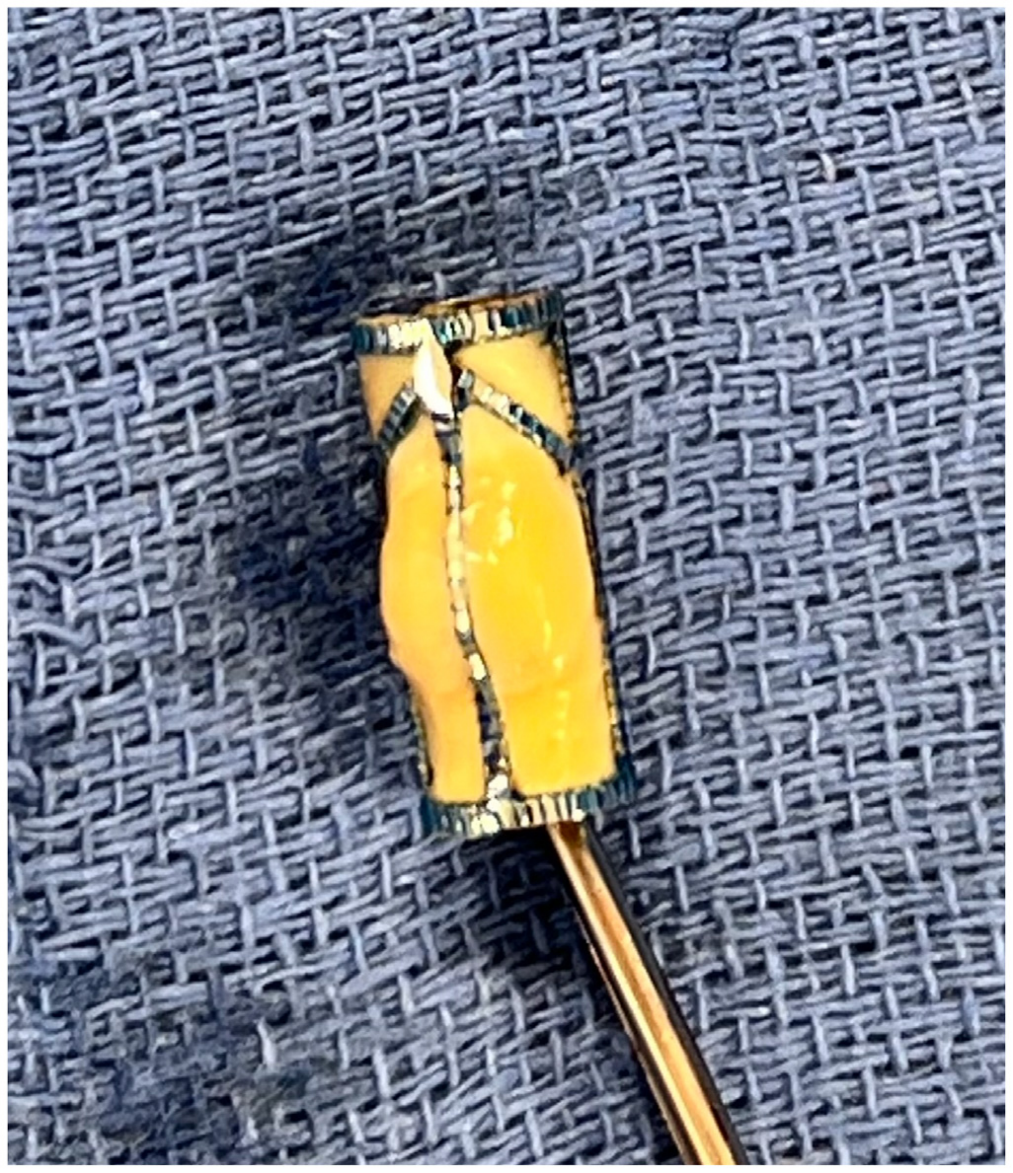
Intraoperative photo of VenoValve device immediately before intravenous implantation.

## Case report

The patient is a 72-year-old man with bilateral venous insufficiency (L > R) and nonhealing wounds on his lower extremities; additional medical history included hypertension. He had previously undergone laser ablation of incompetent saphenous veins and a venogram with intravascular ultrasound examination of his iliac veins to rule out outflow obstruction. He had been undergoing wound care with Unna Boot compression for 4 years without substantial improvement in his symptoms. After informed consent, he was enrolled in the SAVVE study for placement of the VenoValve device. The patient was placed supine on the operating room table and after induction of general anesthesia and normal sterile prepping and draping. A longitudinal incision was made anteriorly over the femoral vein reflecting the adductor longus posteriorly and the femoral sheath was opened allowing the femoral vein and superficial femoral artery to be freed circumferentially. The femoral vein was isolated proximally and distally with silastic loops and clamped 3 minutes after systemic heparinization with profunda clamps. A 1.5-cm venotomy was made and a 10-mm diameter VenoValve device was placed inside the lumen of the vessel, rotating the device into the vein cranially and then brought caudally until the device was centered in the venotomy with the sinus bulge positioned anteriorly. The venotomy was then closed with 6-0 Prolene sutures in a running fashion. Four 6-0 sutures were then used to secure the distal inflow end of the device in place at the 1, 5, 7, and 11 o'clock positions and flow was restored. A sterile ultrasound probe was used to visualize the valve and adjacent femoral vein, which identified no obvious paravalvular leakage or reflux. The wound was closed with 2-0 Vicryl interrupted sutures for fascia, 3-0 Vicryl for deep dermal tissue, and 4-0 Monocryl with skin glue for skin closure. The patient was started on anticoagulation with lovenox for 30 days and then transitioned to apixaban (Eliquis). The device has remained patent at last follow up of 6 months with a decrease in reflux as well as improved Revised Venous Clinical Severity Score and slowly healing ulceration. The patient has been referred to plastic surgery for skin graft placement.

## Conclusions

The VenoValve device is a viable surgical treatment option for patients with CVI who have failed previous conservative and superficial venous interventions. This device has shown excellent efficacy at 2 years, with promise for durable repair beyond that. This procedure is well-tolerated and may serve as an acceptable treatment in properly selected patients. Forthcoming results from the SAVVE study will hopefully provide further information on long-term patency and symptomatic benefits.

## Disclosures

None.
